# *Caballeronia* Bacteremia in Children with Cancer, United States

**DOI:** 10.3201/eid3206.260342

**Published:** 2026-06

**Authors:** Heather L. Glasgow, Sara M. Federico, Raul C. Ribiero, Elisabeth E. Adderson

**Affiliations:** St. Jude Children’s Research Hospital, Memphis, Tennessee, USA (H.L. Glasgow, S.M. Federico, R.C. Ribiero, E.E. Adderson); St. Jude Children’s Research Hospital Graduate School of Biomedical Sciences, Memphis (S.M. Federico, R.C. Ribiero, E.E. Adderson); University of Tennessee Health Science Center, Memphis (S.M. Federico, R.C. Ribiero, E.E. Adderson)

**Keywords:** bacteria, Caballeronia, Burkholderiaceae, bacteremia, cancer, child, pediatric, United States

## Abstract

*Caballeronia* spp. are gram-negative bacteria belonging to the Burkholderiaceae family, commonly found in the environment and in association with plants and animals. However, the bacteria are an uncommon cause of human infection. We describe *Caballeronia* bacteremia in 2 children with cancer in Tennessee, USA.

Burkholderiaceae is a large and heterogeneous bacterial group, ubiquitous in the environment and animal microbiomes, including plant and animal endophytes, nitrogen-fixing symbionts, and plant and animal pathogens (*1*–*3*). Analysis of 16S rRNA and conserved sequence insertion/deletions split the group into 7 distinct clades, including the new genus *Caballeronia*, represented by type species *C. glathei* (*1*–*4*). Named after Mexican microbiologist Jesús Caballero-Mellado, *Caballeronia* are gram-negative nonsporulating oval or rod-shaped cells that occur singly, in pairs, or in short chains. They can grow on tryptone soy and occasionally on MacConkey agar. Whereas members of the *Burkholderia* genus are established human pathogens, *Caballeronia* spp. are not (*2*,*3*). Two strains of *C. concitans* isolated from lung tissue and blood and 1 of *C. turbans* isolated from pleural fluid have been characterized phenotypically, but the clinical details of those or other infections have not been previously reported (*1*,*5*). We describe *Caballeronia* bacteremia in 2 children with cancer in Tennessee, USA.

## The Cases

Case 1 was in a 3-year-old boy with neuroblastoma who was admitted to St. Jude Children’s Research Hospital (Memphis, TN, USA) with a fever of 39.7°C, chills, tachycardia, and hypotension. At the time of hospitalization, he had completed chemotherapy, had undergone resection of his primary tumor and autologous hematopoietic stem-cell transplant, and was receiving radiation therapy to his abdomen. A double-lumen tunneled central venous catheter (CVC) had been placed 15 months earlier. He had a history of catheter-associated bloodstream infections caused by *Escherichia coli* and *Staphylococcus aureus* bacteremia, both successfully treated with antibacterial drugs.

His physical examination was remarkable for pallor, an ill appearance, tachycardia (heart rate 192 beat/min), hypotension (blood pressure 63/51 mm Hg), and a delayed capillary refill of 4–5 seconds. Physical examination showed no focal findings. His leukocyte count was 2.67 × 10^3^ cells/mm^3^ (reference range 5.60–17.00 × 10^3^ cells/mm^3^), and absolute neutrophil count (ANC) was 1,730/mm^3^ (reference range 1,500–8,500/mm^3^). Findings from a complete metabolic panel were unremarkable. Blood cultures were obtained, and intravenous fluids, ceftriaxone, and vancomycin were administered. The patient’s vital signs promptly normalized, and his fever resolved.

Elongated gram-negative bacilli were identified in blood cultures obtained from 1 lumen of the central line after 35.11 incubation and a peripheral vein at 36.17 hours of incubation. Eplex BCID-GN multiplex PCR (Roche, https://www.roche.com) and Accelerate Pheno BC Kit (Accelerate Diagnostics, https://acceleratediagnostics.com) did not identify any organisms. Subcultures grew on blood and chocolate agar but not on MacConkey agar, raising concern for a potential biothreat agent. The Gram stain morphology was consistent with *Burkholderia* spp. but not *Brucella*, *Francisella*, or *Yersinia* spp. Rapid biochemical testing showed the isolate was weakly catalase positive, urea negative, and oxidase positive. A zone of inhibition around a polymyxin B disk ruled out *Burkholderia mallei* and *B. pseudomallei*. Matrix-assisted laser desorption/ionization time-of-flight (MALDI-TOF) mass spectrometry by VITEK MS (bioMérieux, https://www.biomerieux.com) produced no identification. VITEK 2 (bioMérieux) automated biochemical identification cards produced an initial identification of *Burkholderia gladioli* with a confidence score of 89%, which was below the acceptable threshold. Repeat testing from chocolate agar produced no identification but from blood agar produced an identification of *Acinetobacter lwoffii* (confidence score 99%). However, reference laboratory testing (Mayo Clinic Laboratories, Rochester, MN, USA) identified *Caballeronia glathei* by MALDI-TOF mass spectrometry. The identification was confirmed by bacterial whole genome sequencing, as previously described (*6*), and species identification with SpeciesFinder-2.0 (Center for Genomic Epidemiology, https://cge.food.dtu.dk/services/SpeciesFinder). The isolate failed to grow on antimicrobial susceptibility test media.

Additional blood cultures obtained on the second and fourth hospital days were sterile, as was a culture of the patient’s urine. The CVC was retained because the differential time-to-positivity suggested it was not a source of infection. Vancomycin was discontinued after 48 hours, and the patient completed a 10-day course of ceftriaxone.

Case 2 was in a 6-year-old boy with blastic plasmacytoid dendritic cell neoplasm who was admitted to St. Jude Children’s Research Hospital with a history of a fever of 38.2°C at home and loose stools. He was receiving an individualized chemotherapy protocol and had no prior complications from that therapy. A double-lumen CVC had been placed 5 months previously. On evaluation, he was afebrile and well-looking. He received a dose of ceftriaxone and was discharged but was recalled and admitted to hospital the following day when growth was detected in blood cultures (both lumens, at 19.10 and 26.42 hours of incubation). No concurrent blood cultures from a peripheral vein were obtained.

Physical examination at admission was unremarkable, and he had no complaints. His leukocyte count was 1.2 × 10^3^ cells/mm^3^ (reference range 4.40–10.60 × 10^3^ cells/mm^3^) and ANC was 1,281/mm^3^ (reference range 1,500–7,600/mm^3^). Findings of a complete metabolic panel were unremarkable. Additional blood cultures were obtained and cefepime was administered. Later that day, fever recurred (38.1°C), hypotension (blood pressure 82/45 mm Hg) and neutropenia (ANC 334/mm^3^) were noted, and meropenem and trimethoprim/sulfamethoxazole (TMP/SMX) were substituted for cefepime. The patient’s vital signs promptly normalized.

A blood culture broth smear revealed gram-negative bacilli with tapered ends and possible endospores (Figure). Microbiologic characteristics were as described for case 1, with no growth on MacConkey agar. Rapid biochemical testing was negative for oxidase, weakly positive for catalase, and urea positive. After biothreat was ruled out, the reference laboratory testing identified the organism as *Caballeronia* spp. (undetermined species) by 16S ribosomal RNA gene sequencing. The isolate was susceptible to all tested antimicrobial agents, including piperacillin/tazobactam (MIC <8/4 µg/mL), aztreonam (MIC <4 µg/mL), ceftazidime (MIC <4 µg/mL), cefepime (MIC <2 µg/mL), meropenem (MIC 1 µg/mL), ciprofloxacin (MIC 0.5 µg/mL), levofloxacin (MIC <0.5 µg/mL), tobramycin (MIC <1 µg/mL), gentamicin (MIC <1 µg/mL), amikacin (MIC <1 µg/mL), and TMP/SMX (MIC <0.5/9.5 µg/mL).

**Figure:**
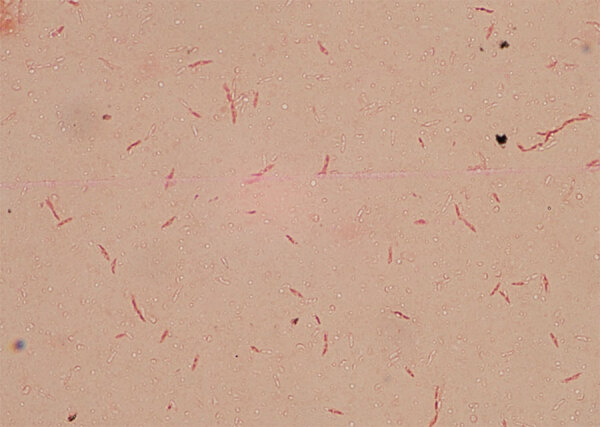
Gram stain of isolate from case 2 in report of 2 cases of *Caballeronia* bacteremia in children with cancer, United States. Staining shows a gram-negative bacillus in pairs and chains with tapered ends and possible endospores. Original magnification ×100.

Subsequent blood cultures were positive for the same organism on the patient’s second hospital day (1 lumen only); cultures were sterile thereafter. The CVC was retained because of the patient’s prompt clinical response and the rapid sterilization of blood cultures. He completed a 10-day course of meropenem and TMP/SMX. Bacterial identification and antimicrobial susceptibility test results were not available until antimicrobial therapy was completed.

## Conclusions

A clear source of these patients’ infections was not found. Physical examination did not reveal a focus of infection that could have resulted in bacteremia. Differential time-to-positivity suggested the second patient had a catheter-related bloodstream infection; contamination of medical devices by Burkholderiaceae has been reported (*7*). Given that the human gastrointestinal microbiome includes *Caballeronia*, infection might have been related to gastrointestinal barrier dysfunction and translocation of bacteria, a route suggested by diarrhea in case 2. Consistent with that hypothesis, some patients with COVID-19 and gastrointestinal symptoms were reported to have elevated markers of gut permeability and plasma microbiomes that contain *Caballeronia* spp. and other gut flora (R. Prasad et al., unpub. data, https://doi.org/10.1101/2021.04.06.438634). Because the 2 cases occurred years apart, healthcare-associated transmission was not suspected.

As in the cases we describe, identification of *Caballeronia* spp. is unlikely to be made with current standard methods available in most clinical laboratories, and misidentification as other *Burkholderia* or *Acinetobacter* spp. could occur. *Caballeronia* spp. are absent from current VITEK MS (bioMérieux) or MALDI Biotyper (Bruker Daltonics) Food and Drug Administration–cleared databases; however, they can be identified by MALDI-TOF mass spectrometry with a custom database (*8*).

The optimal treatment for infections caused by *Caballeronia* spp. is unknown. Administration of broad-spectrum antimicrobial drugs led to clinical and microbiological resolution of the patients’ infections; in case 2, in vitro susceptibility to all antimicrobial drugs tested was confirmed. Of note, both patients’ ANC results were within reference ranges or transiently low; the absence of severe neutropenia might have contributed to a successful outcome. This report of 2 *Caballeronia* bacteremia cases serves as a reminder that emerging pathogens continue to pose clinical challenges in severely immunocompromised patients.
